# Enhanced nitrogen use efficiency, growth and yield of wheat through soil urea hydrolysis inhibition by *Vachellia nilotica* extract

**DOI:** 10.3389/fpls.2022.1039601

**Published:** 2022-11-14

**Authors:** Muhammad Ajmal Rana, Rashid Mahmood, Faisal Nadeem, Yun Wang, Chongwei Jin, Xingxing Liu

**Affiliations:** ^1^ Department of Agronomy, University of the Punjab, Lahore, Pakistan; ^2^ Department of Soil Science, University of the Punjab, Lahore, Pakistan; ^3^ Center of Planting Technology Extension of Dongyang, Jinhua, China; ^4^ State Key Laboratory of Plant Physiology and Biochemistry, College of Natural Resources and Environmental Science, Zhejiang University, Hangzhou, China

**Keywords:** *Vachellia nilotica* extract, hydroquinone, urea, urease inhibition, nitrogen use efficiency, wheat

## Abstract

Soil urease inhibition slows down the urea hydrolysis and prolongs nitrogen (N) stay in soil, resulting in an increased N uptake by plants. Apart from several chemical urease inhibitors, the urease inhibition potential of plant extracts is rarely reported. In our previous study, the soil urease inhibition by *Vachellia nilotica* leaf extract was reported; however, its role in relation to growth and yield of wheat (*Triticum aestivum*) under pot and field conditions remains unknown. The acetonic extracts of 10, 20, and 50 g *Vachellia nilotica* leaves were given code names *viz.* Vn.Fl-10, Vn.Fl-20 and Vn.Fl-50, respectively, and coated on 100 g of urea individually. The enhancements of growth (total number of tillers, number of productive tillers, number of spikelets per spike, number of grains per spike, and 1000-grains weight) and yield (biological yield, straw yield, and grain yield) parameters of wheat by Vn.Fl-20 and Vn.Fl-50 coated urea treatments were compared with uncoated urea in a pot experiment. The experiment indicated that the *Vachellia nilotica* extract coatings were effective at improving N persistence in soil, as reflected by increased grain and straw N concentrations as well as uptakes. The reproduction of the aforementioned results, at the half and full recommended dose of urea under field conditions, reconfirmed the effectiveness of *Vachellia nillotica* coatings. Moreover, the Vn.Fl-20 and Vn.Fl-50 coated urea, at the half as well as full recommended dose under field conditions, proved equally effective in terms of higher biological, straw, and grain yield, and grain N uptake. The increments in the total number of tillers, number of productive tillers, 1000-grain weight, biological yield, straw yield, grain yield, grain N concentration, grain N-, and straw N uptake along with nitrogen use efficiency (NUE) components, i.e. nitrogen partial factor productivity (NPFP), nitrogen agronomic efficiency (NAE), partial nitrogen balance (PNB), and nitrogen recovery efficiency (NRE) of wheat highlighted the superiority of Vn.Fl-20 coating over the hydroquinone (Hq) coating on urea at the full recommended dose under field conditions. Given the findings of this study, *Vachellia nilotica* leaf extract coating (Vn.Fl-20) can be used as a natural urease inhibitor to reduce urea hydrolysis and enhance wheat productivity.

## Introduction

Nitrogen (N), one of the essential primary macronutrients, is needed for the proper functioning of chlorophyll biosynthesis, photosynthesis, carbon assimilation, and protein synthesis processes in terrestrial plants (Leghari et al., 2016). Considering the high N concentration (46%), water solubility, and ease of use, urea is the widely used source of N accounting for almost 50% of the total N consumption worldwide (Motasim et al., 2021; Tanan et al., 2021). However, urea is hydrolyzed to ammonia and carbon dioxide in soil, whereas the presence of urease enzyme escalates this process 8 × 10^7^ times (Rana et al., 2021). Most of the soils have high urease activity that results in rapid production and release of ammonia from the applied urea fertilizer (Dejarmakeerthi and Thenabadw, 1996). According to an estimate, 20 to 70% of urea is lost from soils through ammonia volatilization (Suherman and Anggoro, 2011; Naz and Sulaiman, 2016). The crop plants recover as low as 30-40% N from fertilizer sources, like urea, and the value becomes even lower upon the losses through continuous urea hydrolysis (Shi et al., 2012; Soares et al., 2012). Hence, the use of urease inhibitor coated urea fertilizer becomes even more vital for the reduction of N losses through urea hydrolysis (Artola et al., 2011; Li et al., 2017). The inhibition of soil ureases results in a decrease in ammonia volatilization losses from soil-applied urea (Bishop and Manning, 2010).

The compounds with urease inhibition potential were largely developed to inhibit urease in the human stomach by disinfecting *Helicobacter pylori.* However, their use might not be suitable or economical for soil urease inhibition (Kiss and Simihăian, 2002). Among soil urease inhibitors, N-(n-butyl) thiophosphoric triamide (NBPT) is known for its high urease inhibition potential and non-toxicity to plants (Silva et al., 2017) with few limitations. Firstly, NBPT degrades over time and the addition of organic amendments in the field reduces its shelf life (Watson et al., 2008). Secondly, NBPT-induced decrease in NH_3_ loss is dependent on soil type and temperature (Trenkel, 2010). Similarly, hydroquinone (Hq) is another commercially available synthetic urease inhibitor that may prove phytotoxic as reported in cowpea (Mensah et al., 2012) and rice (Pandey et al., 2005). Being a benzene metabolite, Hq may also prove haematotoxic and carcinogenic (Enguita and Leitão, 2013).

Plant materials that have urease inhibition potentials are an alternative to synthetic urease inhibitors. The list of plant materials that have been tested for soil urease inhibition is rather short, with *Azadirachta indica, Acacia caven*, *Quillaja saponaria*, *Bacharis linearis*, *Pinus radiata*, and *Mentha spicata* the most promising candidates (Patra et al., 2006; Suescun et al., 2012; Mathialagan et al., 2017). In our previous study, we tested the extracts of 35 plant materials for their potential to inhibit jack bean and soil ureases. In this regard, the extract of *Vachellia nilotica* leaves was found to be the best at inhibiting jack bean as well as soil ureases and prolonging the urea-N stay in the soil (Rana et al., 2021). In this study, various concentrations of *Vachellia nilotica* extract coatings on urea, at the half as well as a full dose of recommended urea-N fertilization, were tested in terms of their impact on growth, yield and nitrogen use efficiency (NUE) of wheat cultivated under pot and field conditions.

## Materials and methods

### Preparation of *Vachellia nilotica* extracts and hydroquinone coated urea

Leaves of *Vachellia nilotica* were collected, dried in the shade, and ground using an electric grinder. The powdered material, equivalent to 10, 20, and 50g of fresh leaves, was extracted using 100 ml of acetone by shaking at 200 rpm for 48 hours followed by filtration through Whatman filter paper grade 1. The filtrates were concentrated to about 3 ml by solvent evaporation at 70°C (Rana et al., 2021). For the coating of urea, concentrated extracts of 10, 20, and 50 g of *Vachellia nilotica* leaves were poured over 100 g of urea prills rotating in a rotary mixer till uniform application of the extract over the surface of the prills. The coated urea prills were then removed from the rotary mixer container, dried in shade, and reweighed to quantify the weight of 10, 20, and 50 g of *Vachellia nilotica* leaf extract coatings on respective 100 g urea prills. Thus, the quantities of 10, 20, and 50 g of *Vachellia nilotica* leaf extract coatings were quantified to be 1.2, 2.1, and 4.7 g per 100 g of urea prills, respectively. These coatings of 10, 20, and 50 g of *Vachellia nilotica* leaf extract were coded as Vn.Fl-10, Vn.Fl-20 and Vn.Fl-50, respectively.

For the preparation of hydroquinone (Hq) coated urea, 1 g of Hq was dissolved in the minimum amount of acetone. The mixture was then poured over 100 g of urea rotating in the rotary mixer (Wang et al., 1991). After uniform mixing the coated urea was removed and dried in shade.

### Pot experiment for the screening of *Vachellia nilotica* extract coatings

A pot experiment was conducted to screen the three *vachellia nilotica* extract coatings, *viz* Vn.Fl-10, Vn.Fl-20 and Vn.Fl-50, on urea prills in terms of growth, yield, and N status of wheat (Faisalabad-2008 cultivar). Field soil (0-15 cm depth) was collected, passed through a 2 mm sieve, and filled in polythene lined earthen pots with a 25cm length and 15cm diameter. The physical and chemical characteristics of the soil are given in **Table 1**. Ten seeds of wheat cultivar were sown per pot and three seedlings per pot were maintained at the two-leaves stage after the germination.

**Table 1:** Physical and chemical properties of soils.

Parameters	Trials		
	Pot trial	1^st^ field trial	2^nd^ field trial
Sand (%)	21.0	21.6	21.6
Silt (%)	58.5	58.2	58.2
Clay (%)	20.5	20.2	20.2
Textural class	Silt loam	Silt loam	Silt loam
Organic matter (%)	0.59	0.61	0.62
pH	7.8	7.8	7.9
Electrical conductivity (dS m^-1^)	2.60	2.64	2.69
CEC (cmol_c_ kg^-1^)	6.3	6.5	6.4
Total N (%)	0.031	0.032	0.033
Available P (mg kg^-1^)	6.50	6.46	6.40
Extractable K (mg kg^-1^)	168	165	159
Urease activity (µg N g^-1^ soil 2h^-1^)	102	108	111

CEC, Cation exchange capacity; N, Nitrogen; P, Phosphorus; K, Potassium.

Four treatments were given to the uncoated urea and the Vn.Fl-10, Vn.Fl-20, and Vn.Fl-50 coated urea, providing 120 kg N ha^-1^. The treatments were applied in three replicates under a completely randomized design (CRD). Phosphorus (90 kg ha^-1^) as single super phosphate, potassium (60 kg ha^-1^) as sulfate of potash, and half of the nitrogen were applied as basal dose to uncoated or coated urea. The remaining half dose of urea-N, as uncoated or coated urea, was applied 30 days after the germination. Pots were irrigated with canal water, keeping the moisture level of the soil at or near the field capacity. Weeds were eradicated manually.

Parameters like plant height, the number of total tillers, the number of productive tillers, spike length, the number of spikelets per spike, the number of grains per spike, 1000-grain weight, biological yield, straw yield, and grain yield were determined during growth period or at the final harvest. The concentrations of N in grains and straw were determined by digesting the grain and straw samples, using H_2_SO_4_-H_2_O_2_, followed by the distillation through the Kjeldahl apparatus (Baker and Thompson, 1992). N uptake in grains and straw was calculated by multiplying grain and straw yields with respective N concentrations (Belete et al., 2018).

### Field experiment to determine the performance of *Vachellia nilotica* extract coated urea at the half and full recommended rate of nitrogen

From the screening pot experiment, the best performing *Vachellia nilotica* extract coatings on urea were selected and further investigated under field conditions at two N levels *viz.* half and full recommended rates of nitrogen. The field trial was conducted in the farm area in which rice-wheat crop rotation had taken place for more than five years at the Faculty of Agricultural Sciences, University of the Punjab, Lahore. On 15 November 2019, the seeds of the wheat cultivar (Faisalabad-2008) were sown through the pora method. After sowing, the field was divided to attain a net plot size of 4.5 × 6.0 m^2^.

The treatments were applied by following the two-factor factorial Randomized Complete Block Design (RCBD) with three replications. The first factor was the rate of nitrogen, i.e. half of the recommended (60 kg N ha^-1^) and fully recommended (120 kg N ha^-1^) N dose applied as urea, whereas the second factor was the type of urea, i.e. uncoated urea and coated urea (Vn.Fl-20 and Vn.Fl-50). The P and K fertilizer rates were the same as described in the screening pot experiment. Half a dose of the N fertilizer, either as coated or uncoated urea, was applied with full doses of P and K as basal application. The remaining half dose of the N in the form of either coated or uncoated urea was applied 30 days after germination.

A quadrant (1m^2^ dimension) was used to make three random selections in each experimental plot. The number of total and productive tillers, surrounded by the quadrant boundary was counted and an average number of tillers per m^2^ was calculated. The data of 10 randomly selected plants from each plot were averaged for the determination of plant height, number of spikelets per spike, and grains per spike. From every plot, the wheat crop was harvested manually from ground level at maturity, weighed to record biological yield, and threshed for grain yield. The straw yield was calculated as a difference between biological yield and grain yield. The N concentration and uptake of wheat grains and straw were determined as described in the preceding screening pot experiment.

### Field experiment for the comparison of the effectiveness of *Vachellia nilotica* extract coated- and hydroquinone coated urea

Based on the results of the preceding field experiment, *Vachellia nilotica* extract coating (Vn.Fl-20) was selected and compared with Hq (a reference urease inhibitor) coatings on urea applied at the full recommended dose (120 kg N ha^-1^) during the year 2021. The experiment comprised four treatments, *viz.* no N application, N application as uncoated urea, Hq coated urea, and Vn.Fl-20 coated urea, applied under the Randomized Complete Block Design (RCBD) with three replications. All other details related to location, plot size, sowing method, fertilizer application method, data collection, and plant sample analysis were the same as described in the preceding field experiment. In addition, various components of N use efficiency (NUE) of the wheat crop were also calculated in this experiment by using the following formulae (Fixen et al., 2015).


$$\begin{array}{cc} NPFP\;=\;\frac{Y_{N}}{N} & \ldots (i)\\ NAE=\frac{\left(Y_{N}-Y_{0}\right)}{N} & \ldots (ii)\\ PNB=\frac{U_{GN}}{N} & \left(iii\right)\\ NRE=\frac{\left(U_{TN}-U_{T0}\right)}{N} & \ldots (iv)\\ IUE=\frac{Y_{N}}{U_{GN}} & \ldots (v)\\ PE=\frac{\left(Y-Y_{0}\right)}{\left(U_{GN}-U_{G0}\right)} & \ldots (vi)\\ EA=\frac{U_{TN}}{N} & \ldots (vii) \end{array}$$

Where: NPFP = Nitrogen partial factor productivity

Y_N_ = Grain yield of urea-N fertilized plot

N = Amount of urea-N applied

NAE = Nitrogen agronomic efficiency

Y_0_ = Grain yield of control plot (no N applied)

PNB = Partial nitrogen balance

U_GN_ = Nitrogen uptake by grains of urea-N fertilized plot

NRE = Nitrogen recovery efficiency

U_TN_ = Total nitrogen uptake (grain plus straw) of urea-N fertilized plot

U_T0_ = Total nitrogen uptake (grain plus straw) of control plot (no N applied)

IUE = Internal utilization efficiency

PE = Physiological efficiency

U_G0_ = Nitrogen uptake by grains of control plot (no N applied)

### Statistical analysis

The data of all the experiments were subjected to analysis of variance (ANOVA) and the means were compared by Tukey’s HSD test (p < 0.05) using Statistix-8.1 software.

## Results

### Effectiveness of different rates of *Vachellia nilotica* extract coatings on urea prills in terms of growth, yield, and N uptake of wheat

Except for plant height and spike length, the growth and yield parameters along with N uptake were significantly influenced by two of the three treatments of *Vachellia nilotica* extract coated urea (Vn.Fl-20 and Vn.Fl-50) as compared to uncoated urea (**Table 2**). In comparison to uncoated urea treatment, the application of Vn.Fl-20 increased the number of total tillers, number of productive tillers, number of spikelets per spike, number of grains per spike, and 1000-grains weight, significantly, by 10, 8, 9, and 6%, respectively. The responses of wheat to Vn.Fl-20 and Vn.Fl-50 coated urea, in terms of plant height, number of total tillers, number of productive tillers, spike length, number of spikelets per spike, number of grains per spike, and 1000-grain weight, were alike. However, the application of Vn.Fl-10 coated urea remained non-significant for uncoated urea (**Table 2** and **Supplementary Table 1**).

**Table 2:** The growth, yield, and N status of wheat supplied with different rates of *Vachellia nilotica* extract coated urea in pot experiments.

Parameters	Treatments			
	Uncoated Urea	*Vn.Fl-10	*Vn.Fl-20	*Vn.Fl-50
Plant height (cm)	71.72	72.15	72.63	73.08
Total no. of tillers	7.38 b	7.55 b	8.00 a	8.17 a
No. of productive tillers	6.78 b	6.83 b	7.22 a	7.39 a
Spike length (cm)	10.32	10.43	10.47	10.52
No. of spikelets per spike	14.84 c	15.17 bc	16.05 ab	16.25 a
No. of grains per spike	38.62 b	39.11 b	40.47 a	41.03 a
1000-grains weight (g)	38.4 b	38.9 b	41.1 a	42.1 a
Biological yield (g pot^-1^)	50.12 c	51.52 bc	53.89 ab	55.17 a
Straw yield (g pot^-1^)	29.25 b	30.34 ab	31.39 a	32.09 a
Grain yield (g pot^-1^)	20.87 c	21.18 bc	22.50 ab	23.08 a
Grain N concentration (%)	2.08 b	2.12 b	2.22 a	2.24 a
Straw N concentration (%)	0.46 b	0.49 b	0.56 a	0.59 a
Grain N uptake (g pot^-1^)	0.41 b	0.43 b	0.48 a	0.50 a
Straw N uptake (g pot^-1^)	0.09 b	0.10 b	0.12 a	0.13 a

*100 g of urea coated with the extract of 10 g (Vn.Fl-10), 20 g (Vn.Fl-20) and 50 g (Vn.Fl-50) fresh leaves of Vachellia nilotica. Values are the means of three replicates. Different letters indicate significance at p < 0.05.

Similarly, Vn.Fl-20 treatment enhanced the biological, straw, and grain yields, significantly, by 7, 7, and 8%, respectively, for uncoated urea (**Table 2** and **Supplementary Table 1**). The application of Vn.Fl-50 did not enhance the aforementioned parameters, significantly, with respect to Vn.Fl-20. Likewise, the performance of Vn.Fl-10 proved to be statistically non-significant as compared to uncoated urea and Vn.Fl-20 (**Table 2**).

The Vn.Fl-20 and Vn.Fl-50 coated urea treatments increased grain N concentration (7 and 8%), straw N concentration (22 and 28%), grain N uptake (17 and 22%), and straw N uptake (33 and 44%), respectively for wheat in comparison to uncoated urea (**Table 2** and **Supplementary Table 1**). Like the growth and yield parameters, the N status of wheat plants treated with Vn.Fl-10 remained statistically similar to that of uncoated urea (**Table 2**). Hence, Vn.Fl-20 and Vn.Fl-50 was found to have a significantly positive impact, as *Vachellia nilotica* extract coated urea treatments, on growth, yield, and N status of wheat (**Table 2**).

### Growth, yield, and N status of field grown wheat under Vn.Fl-20 and Vn.Fl-50 coated urea applications

*Vachellia nilotica* extract coated urea treatments, Vn.Fl-20 and Vn.Fl-50, showed statistically similar results, though significantly higher than those of uncoated urea treatment, for all the studied parameters (plant height, number of total tillers, number of productive tillers, spike length, number of spikelets per spike, number of grains per spike, 1000-grains weight, biological yield, straw yield, grain yield, grain N concentration, straw N concentration, and straw N uptake). Nonetheless, grain N uptake was significantly higher in wheat under Vn.Fl-50 than that of Vn.Fl-20 treatment (**Table 3**). There were significant increments observed in plant height (3 and 4%), the total number of tillers (6 and 7%), number of productive tillers (5 and 7%), spike length (2 and 4%), number of spikelets per spike (2 and 3%), number of grains per spike (6 and 7%) and 1000-grains weight (9 and 10%) of wheat under Vn.Fl-20 and Vn.Fl-50 treatments, respectively, in comparison to uncoated urea (**Supplementary Table 3**). Likewise, the biological, straw, and grain yields were also enhanced by 16, 17, and 13% under Vn.Fl-20 treatment, and 17, 18, and 15% under Vn.Fl-50 treatment, respectively, as compared to uncoated urea (**Supplementary Table 3**). Besides the abovementioned growth and yield attributes, Vn.Fl-20 and Vn.Fl-50 significantly enhanced the grain N concentration (8 and 115%), straw N concentration (18 and 24%), grain N uptake (22 and 26%), and straw N uptake (36 and 43%), respectively, as compared to uncoated urea (**Table 3** and **Supplementary Table 3**).

**Table 3:** The growth, yield, and N status of wheat supplied with different rates of *Vachellia nilotica* extract coated urea in field conditions (2019-20).

Parameters	Treatments		
	Uncoated Urea	*Vn.Fl-20	*Vn.Fl-50
Plant height (cm)	91.23 b	94.28 a	95.19 a
Total no. of tillers m^-2^	387.16 b	409.45 a	416.31 a
No. of productive tillers m^-2^	381.74 b	399.82 a	408.78 a
Spike length (cm)	10.12 b	10.36 a	10.51 a
No. of spikelets per spike	15.62 b	15.98 a	16.11 a
No. of grains per spike	42.20 b	44.82 a	45.03 a
1000-grains weight (g)	40.81 b	44.41 a	45.11 a
Biological yield (t ha^-1^)	10.20 b	11.80 a	11.93 a
Straw yield (t ha^-1^)	5.78 b	6.79 a	6.84 a
Grain yield (t ha^-1^)	4.42 b	5.01 a	5.08 a
Grain N concentration (%)	2.14 b	2.32 a	2.37 a
Straw N concentration (%)	0.51 b	0.60 a	0.63 a
Grain N uptake (kg ha^-1^)	95.53 c	116.74 b	120.89 a
Straw N uptake (kg ha^-1^)	30.19 b	41.15 a	43.10 a

*100 g of urea coated with the extract of 20 g (Vn.Fl-20) and 50 g (Vn.Fl-50) fresh leaves of Vachellia nilotica. Values are the means of three replicates. Different letters indicate significance at p < 0.05.

The interaction effects of type of urea (uncoated, Vn.Fl-20 and Vn.Fl-50) and urea-N dose (half and full of the recommended dose) were non-significant (p < 0.05) for all the studied parameters, except biological yield, grain yield, straw yield and grain N uptake of wheat. The interaction effects of type of urea and urea-N dose for biological yield, grain yield, straw yield, and grain N uptake were significant, having minimum values under uncoated urea application while maximum values under *Vachellia nilotica* extract coated urea application (either as Vn.Fl-20 or as Vn.Fl-50 treatment) under half as well as full recommended rates of N. Specifically, the application of Vn.Fl-20 at half of the recommended rate of N increased biological yield, straw yield, grain yield, and grain N uptake, on average, by 21, 22, 21, and 34%, respectively, whereas Vn.Fl-50 increased the same parameters by 12, 14. 10 and 18%, respectively, but at the full recommended rate of N (**Table 4**). Talking about the main effects, in comparison to its half dose, the full recommended dose of N significantly (p < 0.05) improved all growth and yield parameters together with the N status of wheat (**Supplementary Table 2**).

**Table 4:** Interaction effect of *Vachellia nilotica* extract coated urea and rate of urea-N application on Yield and N uptake of wheat under field conditions (2019-20).

Urea Rate	Treatments	Biological yield (t ha^-1^)	Straw yield(t ha^-1^)	Grain yield(t ha^-1^)	Grain N uptake (kg ha^-1^)
Half of recommended Urea-N	Uncoated Urea	9.01 d	5.17 c	3.84 d	76.30 d
	*Vn.Fl-20	10.83 c	6.27 b	4.56 c	100.45 c
	*Vn.Fl-50	10.96 c	6.33 b	4.63 c	104.46 c
Full recommended Urea-N	Uncoated Urea	11.39 b	6.39 b	5.00 b	114.77 b
	*Vn.Fl-20	12.76 a	7.31 a	5.45 a	133.02 a
	*Vn.Fl-50	12.89 a	7.35 a	5.54 a	137.33 a

*100 g of urea coated with the extract of 20 g (Vn.Fl-20) and 50 g (Vn.Fl-50) fresh leaves of Vachellia nilotica. Values are the means of three replicates. Different letters indicate significance at p < 0.05.

### Comparative effect of *Vachellia nilotica* extract (Vn.Fl-20) and hydroquinone coated urea on growth and yield parameters of field grown wheat

The results of the application of *Vachellia nilotica* extract coated urea (Vn.Fl-20 and Vn.Fl-50) in the preceding field trial distinctly clarified the significant upregulation of growth, yield, and N status of wheat for uncoated urea either at half or full recommended N dose. In this experiment, the efficacy of *Vachellia nilotica* extract coating (Vn.Fl-20) was determined parallel to Hq coating on urea. Based on the result of previously mentioned screening and field trials of this study, *Vachellia nilotica* extract coated urea treatment Vn.Fl-20 was selected to compare with Hq coated urea with respect to uncoated urea and no urea treatments (**Table 5**). The results indicated that, except for biological yield, the Hq coated urea did not enhance growth and yield parameters as well as the N status of wheat, significantly, in comparison to uncoated urea (**Table 5**). Moreover, the results of Hq coated urea, in terms of growth and yield parameters as well as the N status of wheat, proved to be either non-significant or lower than those of Vn.Fl-20 coated urea application (**Table 5**). On the other hand, the application of Vn.Fl-20 coated urea, significantly, increased the total number of tillers, number of productive tillers, and 1000-grains weight by 10, 11, and 9%, respectively, as compared to Hq coated urea (**Table 5** and **Supplementary Table 4**). Furthermore, Vn.Fl-20 treatment showed increments in biological, grain, and straw yield of wheat by 10, 11, and 9%, respectively, as compared to Hq coated urea, and 14, 16 and 11%, respectively, as compared to uncoated urea (**Table 5**). In addition, Vn.Fl-20 also enhanced grain N concentration, grain N uptake, and straw N uptake by 8, 17, and 18%, respectively, in contrast to Hq coated urea (**Table 5** and **Supplementary Table 4**).

**Table 5:** Comparative effect of *Vachellia nilotica* extract and hydroquinone (Hq) coated urea on the growth, yield, and N status of wheat under field conditions (2020-21).

Parameters	Treatments			
	No Urea (Control)	Uncoated Urea	*Hq coated Urea	*Vn.Fl-20
Plant height (cm)	88.26 c	95.11 b	96.07 ab	98.19 a
Total no. of tillers m^-2^	330.00 c	448.33 b	460.33 b	505.33 a
No. of productive tillers m^-2^	305.00 c	432.67 b	446.00 b	496.33 a
Spike length (cm)	9.43 b	10.62 a	10.65 a	10.87 a
No. of spikelets per spike	15.00 b	16.13 a	16.43 a	16.73 a
No. of grains per spike	37.70 c	45.37 b	46.40 ab	48.73 a
1000-grains weight (g)	33.50 c	44.63 b	45.48 b	49.42 a
Biological yield (t ha^-1^)	7.90 d	11.89 c	12.29 b	13.55 a
Straw yield (t ha^-1^)	4.72 c	6.67 b	6.96 b	7.73 a
Grain yield (t ha^-1^)	3.12 c	5.22 b	5.33 b	5.82 a
Grain N concentration (%)	1.40 c	2.31 b	2.33 b	2.51 a
Straw N concentration (%)	0.33 b	0.62 a	0.65 a	0.69 a
Grain N uptake (kg ha^-1^)	43.72 c	120.35 b	124.30 b	145.84 a
Straw N uptake (kg ha^-1^)	15.68 c	41.56 b	45.33 b	53.39 a

*100 g of urea coated with the extract of 20 g (Vn.Fl-20) fresh leaves of Vachellia nilotica. *Hq; Hydroquinone. Values are the means of three replicates. Different letters indicate significance at p < 0.05.

To further dissect the effectiveness of *Vachellia nilotica* extract coated urea application, various components of nitrogen use efficiency (NUE), *viz*. N partial factor productivity (NPFP), N agronomic efficiency (NAE), partial nitrogen balance (PNB), and N recovery efficiency (NRE) of wheat were calculated (**Table 6**). The results revealed 12, 29, 21, and 36% gains in NPFP, NAE, PNB, and NRE, respectively, of wheat under Vn.Fl-20 coated urea treatment compared to uncoated urea (**Table 6** and **Supplementary Table 4**). Likewise, Vn.Fl-20 coated urea treatment also enhanced the NPFP (9%), NAE (22%), PNB (17%), and NRE (26%) of wheat significantly in the Hq coated urea (**Table 6** and **Supplementary Table 4**).

**Table 6:** Comparative effect of *Vachellia nilotica* extract and hydroquinone (Hq) coated urea on the components of nitrogen use efficiency (NUE) of wheat under field conditions (2020–21).

Parameters	Treatments		
	Uncoated Urea	Hq coated Urea	*Vn.Fl-20
NPFP (kg grain/kg N)	43.49 b	44.44 b	48.49 a
NAE (kg grain/kg N)	17.46 b	18.41 b	22.46 a
PNB (kg N uptake/kg N)	1.00 b	1.03 b	1.21 a
NRE (kg N uptake/kg N)	0.85 c	0.92 b	1.16 a
IUE (kg grain/kg N uptake)	43.36 a	42.90 b	39.90 b
PE	27.34	27.41	26.40

NPFP, Nitrogen partial factor productivity; NAE, Nitrogen agronomic efficiency; PNB, Partial nitrogen balance; NRE, Nitrogen recovery efficiency; IUE, Internal utilization efficiency; PE, Physiological efficiency.

*100 g of urea coated with the extract of 20 g (Vn.Fl-20) fresh leaves of Vachellia nilotica. Values are the means of three replicates. Different letters indicate significance at p < 0.05.

## Discussion

Nitrogen (N) losses, driven by the activities of soil ureases in urea applied agricultural soils, result in poor crop growth and yield along with excessive N deposition, both in freshwater bodies and atmospheric air, causing various environmental pollution risks (Sigurdarson et al., 2018). Previous studies have reported soil urease inhibition using chemical compounds, like N-(n-butyl) thiophosphoric triamide (NBPT), agrotain, and hydroquinone (Hq), etc. as urease inhibitors (Khan et al., 2013; Silva et al., 2017). However, studies reporting the use of plant extracts, like *Vachellia nilotica* extract, in various coating rates in comparison to chemical urease inhibitors (Hq) have rarely been reported to date.

Our previous study reported that *Vachellia nilotica* is a natural inhibitor of urea hydrolysis (Rana et al., 2021). In this study, we further dissected the urease inhibition potential of *Vachellia nilotica* extract by determining its effective dose for urea coatings and subsequent verifications through the investigations of wheat growth, yield, and N status, in field trials in comparison to Hq, a commercially available chemical urease inhibitor. In the initial pot experiment, Vn.Fl-20 and Vn.Fl-50 proved to be the best treatment, which induced significant increases in the total number of tillers, number of productive tillers, number of spikelets per spike, number of grains per spike, and 1000-grain weight (**Table 2**). These enhancements in agronomic parameters by Vn.Fl-20 and Vn.Fl-50 applications were further replicated in the significant gains in biological, straw, and grain yields of wheat (**Table 2**). The aforementioned improvements in growth and yield parameters of wheat, by two of the three, applied *Vachellia nillotica* extract coated urea treatments (i.e. Vn.Fl-20 and Vn.Fl-50), was the result of the inhibition of urea hydrolysis by *Vachellia nillotica* extract coating, with involved prolonged use of fertilizer-N in the soil to enable plant uptake, which might otherwise have been lost through ammonia volatilization (Rana et al., 2021). This argument was also supported by the significantly higher grain N concentration, straw N concentration, grain N uptake, and straw N uptake in wheat under the application of Vn.Fl-20 as well as Vn.Fl-50 (**Table 2**). These increases in N concentrations and uptake could have strengthened the photosynthetic framework of wheat and ensured better carbon-to-nitrogen metabolism for efficient protein and carbon skeleton accumulation (Zhou et al., 2016; Belete et al., 2018; Iqbal et al., 2020), as indicated by the increased biomass and grain yield production (**Table 2**).

Considering the results of the initial screening experiment, the two best performing treatments of *Vachellia nillotica* extract coatings on urea, *viz*. Vn.Fl-20 and Vn-Fl-50, were applied to field grown wheat crops for the determination of their urease inhibition potential under field conditions. Consistent with the findings of the initial screening pot experiment, both Vn.Fl-20 and Vn-Fl-50 significantly enhanced plant height, the total number of tillers, number of productive tillers, spike length, number of spikelets per spike, 1000-grain weight, biological yield, straw yield, grain yield, grain N concentration, straw N concentration, grain N uptake and straw N uptake of wheat in comparison to uncoated urea application under field conditions (**Table 3**). Although the urease inhibition responsive growth and yield benefits to the wheat crop are not reported in the literature (Li et al., 2015), this could have been influenced by the differences in soil N status and application rates of fertilizer N. A crop cultivated on N fertilized soil or nourished well with an excess amount of N would, obviously, show either no or less response to prolonged urea N stay due to urease inhibition (Zhang et al., 2012). Besides the urea hydrolysis inhibition, the extract coatings could also act as a minor physical barrier in the release of N from coated urea granules. However, it might not be true in the case of *Vachellia nilotica* extract coatings due to the hydrophilic and water-soluble nature of the extract. Furthermore, the preparation of slow-release fertilizers mostly requires hydrophobic coating materials (Naz and Sulaiman, 2016). The role of coated fertilizers in the slow release of nutrients is a proven fact and their impact on crop growth and yield parameters is already reported in existing studies (Johnson and Raun, 2003). Although the maximum values of growth and yield parameters of wheat were noted at the recommended rate of N (**Supplementary Table 2**), *Vachellia nilotica* extract coated urea applied at full dose as well as a half dose of the recommended N rate significantly increased biological, straw, and grain yield along with grain N uptake compared to the respective full and half doses of uncoated urea (**Table 4**). The *Vachellia nilotica* extract (Vn.Fl-20 or Vn.Fl-50) coated urea applied at 60 kg N ha^-1^ (half of the recommended dose of urea) achieved an almost similar straw yield compared to uncoated urea applied at 120 kg N ha^-1^ (recommended dose of urea) (**Table 4**). Understandably, the urease inhibitory role of *Vachellia nilotica* extract could have reduced urea losses and increased the availability of N to wheat plants by slowing down the urea hydrolysis in the soil, thus reducing the pool of exchangeable NH_4_^+^, lowering NH_3_ losses (>50%) and enhancing nitrogen use efficiency (Sanz-Cobena et al., 2011; Soares et al., 2012; Khan et al., 2013; Silva et al., 2017). Furthermore, at a half dose of urea application, the wheat plants would have faced N deficiency and, thus, the N saved through urease inhibition proved to be of more utility to them as compared to those wheat plants, which nourished well under a full dose of recommended N supply (Ghafoor et al., 2021).

In the third field experiment, the *Vachellia nilotica* extract coated urea (Vn.Fl-20) proved to be significantly more effective than Hq coated urea in terms of enhancements in growth, yield, and N status of wheat plants (**Table 5**). These enhancements further ensured increments in the NPFP, NAE, PNB, and NRE of wheat as compared to Hq coated urea as well as uncoated urea applications (**Table 6**). As NUE depends on the tradeoff between N demand and the N released from applied fertilizer. The gains in the NPFT, NRE, and PNB (NUE components) of wheat under Vn.Fl-20 coated urea were the result of improved N availability (Fixen et al., 2015), somehow, through slow urea hydrolysis leading to higher biomass and yield production per unit of applied urea-N as compared to that of uncoated urea (Zhang et al., 2012). On the other hand, the internal utilization efficiency (IUE) is defined as the grain yield in relation to the amount of N taken up. A better N uptake requires the controlled release of N from the fertilizer source. A high release of N does not need to ensure higher N uptake by plants. In the case of low N uptake, the IUE remains high (Shewry, 2007; Hawkesford, 2014), as was the case in our study under uncoated urea treatment (**Table 6**). Physiological efficiency (PE) expresses the yield increase in relation to the increase in crop N uptake. Accumulation and distribution of N in the vegetative and reproductive organs of crop plants are important processes for determining PE. In this study, PE remained non-significant in either Vn.Fl-20 coated or uncoated urea treatments (**Table 6**), perhaps, due to the dynamic characteristics of N uptake by wheat plants (Zhang et al., 2009; Obi et al., 2022), which should be further investigated.

In our previous study, Hq was used as the reference urease inhibitor for urea hydrolysis in soil up to 50% (Rana et al., 2021). However, in this study, the effect of Hq on growth, yield, N status, and NUE of wheat was either statistically at par or lower than that of Vn-Fl-20 (**Tables 5**, **6**, and **Supplementary Table 4**). We argued that the inhibitory effect of Hq on urea hydrolysis could be of short-term nature as compared to Vn.Fl-20. This argument is further supported by the fact that the application of Hq with urea significantly reduces N_2_O emission in the wheat-rice system but does not increase the grain yield of wheat in comparison to the application of urea alone (Malla et al., 2005). Moreover, the phytotoxic effects of Hq reported in the case of cowpea (Mensah et al., 2012) and rice (Pandey et al., 2005), could have also reduced its effectiveness in comparison to Vn.Fl-20. Furthermore, Hq, being a benzene metabolite, possesses haematotoxic and carcinogenic characteristics (Enguita and Leitão, 2013). N-(n-butyl) thiophosphoric triamide (NBPT) is reported to interfere heavily with urea-applied N availability in maize plants, which limits the N influx and assimilation (Zanin et al., 2015). Moreover, their effective inhibition potential is a short period with limited shelf life, which calls for the need for further efforts in their research and development (Cantarella et al., 2018). On the other hand, as compared to Hq, in particular, *Vachellia nilotica* leaf extract is a natural product that has minimal environmental and hazardous consequences. It may also prove cost-effective and easy to access, especially, in regions abundantly inhabited by *Vachellia nilotica* trees.

## Conclusion

Given its urea hydrolysis inhibition potential, *Vachellia nilotica* extract coating on urea prills (Vn.Fl.20) enhanced grain N concentration, straw N concentration, grain N uptake, and the straw N uptake of wheat. These conditionings of N, subsequently, resulted in the increased nitrogen partial factor productivity (NPFP), nitrogen agronomic efficiency (NAE), partial nitrogen balance (PNB), and nitrogen recovery efficiency (NRE) of wheat. The increments in the nitrogen use efficiency components by Vn.Fl.20 coated urea, in comparison to both uncoated and hydroquinone (Hq) coated urea under pot as well as field conditions, resulting in the enhancement of the growth and yield attributes of wheat. Hence, *Vachellia nilotica* leaf extract (Vn.Fl-20) can be used as natural urease inhibitor with minimal phytotoxic consequences and maximum soil N retention benefits.

## Data availability statement

The original contributions presented in the study are included in the article/**Supplementary Material**. Further inquiries can be directed to the corresponding authors.

## Author contributions

XL, RM, and MAR conceived the idea and designed the research. MAR, RM, and FN conducted the research. RM and MAR analyzed the data and wrote the paper. FN, YW, CJ, and XL revised the manuscript. All authors approved the final manuscript.

## Funding

The study was supported by the China Postdoctoral Science Foundation (2022T150571), Healthy Soil Projects in DongYang (JCZFCG2022-Z37-C303), and an indigenous Ph.D. Fellowship grant (213-64972-2AV2-162-50025766) by the Higher Education Commission (HEC), Pakistan.

## Conflict of interest

The authors declare that the research was conducted in the absence of any commercial or financial relationships that could be construed as a potential conflict of interest.

## Publisher’s note

All claims expressed in this article are solely those of the authors and do not necessarily represent those of their affiliated organizations, or those of the publisher, the editors and the reviewers. Any product that may be evaluated in this article, or claim that may be made by its manufacturer, is not guaranteed or endorsed by the publisher.
