# Supplementary material for: Enhanced nitrogen use efficiency, growth and yield of wheat through soil urea hydrolysis inhibition by Vachellia nilotica extract
Source: Front Plant Sci. 2022 Nov 14;13:1039601. doi: 10.3389/fpls.2022.1039601 (PMC9702566; doi:10.3389/fpls.2022.1039601)
Supplement: Supplementary file 1 [file DataSheet_1.docx]

**Enhanced nitrogen use efficiency, growth, and yield of wheat through soil urea hydrolysis inhibition by *Vachellia nilotica* extract**

Muhammad Ajmal Rana^1^, Rashid Mahmood^2^, Faisal Nadeem^2,*^, Yun Wang^3^, Chongwei Jin^4^, Xingxing Liu^4^

| **Supplementary Table 1**\| Percent increase in growth, yield and N uptake of wheat as influenced by *Vachellia nilotica* extract coated urea (pot trial) | | | | | | | |
| --- | --- | --- | --- | --- | --- | --- | --- |
| **Parameters** | **Treatments** | | | | | | |
|  | **Actual values** | | | | **Percent increase over uncoated urea (%)** | | |
|  | **Uncoated Urea** | ***Vn.Fl-10** | ***Vn.Fl-20** | ***Vn.Fl-50** | ***Vn.Fl-10** | ***Vn.Fl-20** | ***Vn.Fl-50** |
| Plant height (cm) | 71.72 | 72.15 | 72.63 | 73.08 | 0.60 | 1.27 | 1.90 |
| Total no. of tillers | 7.38 | 7.55 | 8.00 | 8.17 | 2.30 | 8.40 | 10.7 |
| No. of productive tillers | 6.78 | 6.83 | 7.22 | 7.39 | 0.74 | 6.49 | 8.99 |
| Spike length (cm) | 10.32 | 10.43 | 10.47 | 10.52 | 1.06 | 1.45 | 1.94 |
| No. of spikelets per spike | 14.84 | 15.17 | 16.05 | 16.25 | 2.22 | 8.15 | 9.50 |
| No. of grains per spike | 38.62 | 39.11 | 40.47 | 41.03 | 1.27 | 4.79 | 6.24 |
| 1000-grains weight (g) | 38.4 | 38.9 | 41.1 | 4.21 | 1.30 | 7.03 | 9.63 |
| Biological yield (g pot^-1^) | 50.12 | 51.52 | 53.89 | 55.17 | 2.79 | 7.52 | 10.08 |
| Straw yield (g pot^-1^) | 29.25 | 30.34 | 31.39 | 32.09 | 3.73 | 7.32 | 9.71 |
| Grain yield (g pot^-1^) | 20.87 | 21.18 | 22.50 | 23.08 | 1.48 | 7.81 | 10.59 |
| Grain N concentration (%) | 2.08 | 2.12 | 2.22 | 2.24 | 1.92 | 6.73 | 7.69 |
| Straw N concentration (%) | 0.46 | 0.49 | 0.56 | 0.59 | 6.52 | 21.74 | 28.26 |
| Grain N uptake (g pot^-1^) | 0.41 | 0.43 | 0.48 | 0.50 | 4.88 | 17.07 | 21.95 |
| Straw N uptake (g pot^-1^) | 0.09 | 0.10 | 0.12 | 0.13 | 11.11 | 33.33 | 44.44 |
| *****100 g of urea coated with the extract of 10 g (Vn.Fl-10), 20 g (Vn.Fl-20) and 50 g (Vn.Fl-50) fresh leaves of *Vachellia nilotica*. | | | | | | | |

| **Supplementary Table 2**\| Effects of half and full recommended rate of N on growth, yield and N uptake of wheat (field experiment-I). | | | |
| --- | --- | --- | --- |
| **Parameters** | **Half of recommended Urea N** | **Full recommended Urea N** | **HSD** |
| Plant height (cm) | 91.53 b | 95.61 a | 1.24 |
| Total no. of tillers m^-2^ | 385.63 b | 423.0 a | 9.57 |
| No. of productive tillers m^-2^ | 380.56 b | 413 a | 11.0 |
| Spike length (cm) | 10.2 b | 10.47 a | 0.15 |
| No. of spikelets per spike | 15.51 b | 16.30 a | 0.24 |
| No. of grains per spike | 41.93 b | 46.09 a | 0.73 |
| 1000-grains weight (g) | 40.65 b | 46.25 a | 0.55 |
| Biological yield ((kg ha^-1^)) | 10268 b | 12351 a | 98.15 |
| Straw yield ((kg ha^-1^)) | 5923 b | 7019.3 a | 67.62 |
| Grain yield ((kg ha^-1^)) | 4344.8 b | 5331.8 a | 82.35 |
| Grain N concentration (%) | 2.15 b | 2.40 a | 0.07 |
| Straw N concentration (%) | 0.51 b | 0.67 a | 0.03 |
| Grain N uptake (g/pot) | 93.73 b | 128.37 a | 2.05 |
| Straw N uptake (g/pot) | 30.55 b | 47.04 a | 2.04 |
| Grain yield increment (kg ha^-1^) | 494.56 b | 749.64 a | 116.35 |
| Grain uptake increment (kg ha^-1^) | 20.41 b | 26.16 a | 1.86 |
| *****Values are means of three replicates. Different letters indicate significance at p < 0.05 | | | |
|  | | | |

| **Supplementary Table 3**\| Percent increase in growth, yield and N uptake of wheat as influenced by *Vachellia nilotica* extract coated urea under field conditions | | | | | |
| --- | --- | --- | --- | --- | --- |
| **Parameters** | **Treatments** | | | | |
|  | **Actual values** | | | **Percent increase over uncoated Urea (%)** | |
|  | **Uncoated Urea** | ***Vn.Fl-20** | **8Vn.Fl-50** | ***Vn.Fl-20** | ***Vn.Fl-50** |
| Plant height (cm) | 91.23 | 94.28 | 95.19 | 3.34 | 4.34 |
| Total no. of tillers m^-2^ | 387.16 | 409.45 | 416.31 | 5.75 | 7.53 |
| No. of productive tillers m^-2^ | 381.74 | 399.82 | 408.78 | 4.74 | 7.08 |
| Spike length (cm) | 10.12 | 10.36 | 10.51 | 2.37 | 3.85 |
| No. of spikelets per spike | 15.62 | 15.98 | 16.11 | 2.30 | 3.14 |
| No. of grains per spike | 42.20 | 44.82 | 45.03 | 6.21 | 6.71 |
| 1000-grains weight (g) | 40.81 | 44.41 | 45.11 | 8.82 | 10.54 |
| Biological yield (t ha^-1^) | 10.20 | 11.80 | 11.93 | 15.69 | 16.96 |
| Straw yield (t ha^-1^) | 5.78 | 6.79 | 6.84 | 17.47 | 18.34 |
| Grain yield (t ha^-1^) | 4.42 | 5.01 | 5.08 | 13.35 | 14.93 |
| Grain N concentration (%) | 2.14 | 2.32 | 2.37 | 8.41 | 10.75 |
| Straw N concentration (%) | 0.51 | 0.60 | 0.63 | 17.64 | 23.53 |
| Grain N uptake (kg ha^-1^) | 95.53 | 116.74 | 120.89 | 22.2 | 26.55 |
| Straw N uptake (kg ha^-1^) | 30.19 | 41.15 | 43.10 | 36.3 | 42.76 |
| *100 g of urea coated with the extract of 20 g (Vn.Fl-20) and 50 g (Vn.Fl-50) fresh leaves of Vachellia nilotica | | | | | |

| **Supplementary Table 4**\| Percent increase in growth, yield and N uptake of wheat as influenced by *Vachellia nilotica* extract coated and hydroquinone (Hq) coated urea under field conditions | | | | | | |
| --- | --- | --- | --- | --- | --- | --- |
| **Parameters** | **Treatments** | | | | | |
|  | **Actual values** | | | **Percent increase (%)** | | |
|  |  |  |  | **Compared to uncoated Urea** | | **Compared to Hq coated Urea** |
|  | **Uncoated Urea** | **Hq coated Urea** | ***Vn.Fl-20** | **Hq coated Urea** | ***Vn.Fl-20** | ***Vn.Fl-20** |
| Plant height (cm) | 95.11 | 96.07 | 98.19 | 1.01 | 3.24 | 2.21 |
| Total no. of tillers m^-2^ | 448.33 | 460.33 | 505.33 | 2.68 | 12.71 | 9.77 |
| No. of productive tillers m^-2^ | 432.67 | 446.00 | 496.33 | 3.08 | 14.71 | 11.28 |
| Spike length (cm) | 10.62 | 10.65 | 10.87 | 0.28 | 2.35 | 2.06 |
| No. of spikelets per spike | 16.13 | 16.43 | 16.73 | 1.86 | 3.72 | 1.82 |
| No. of grains per spike | 45.37 | 46.40 | 48.73 | 2.27 | 7.40 | 5.02 |
| 1000-grains weight (g) | 44.63 | 45.48 | 49.42 | 1.9 | 10.73 | 8.66 |
| Biological yield (t ha^-1^) | 11.89 | 12.29 | 13.55 | 3.36 | 13.96 | 10.25 |
| Straw yield (t ha^-1^) | 6.67 | 6.96 | 7.73 | 4.35 | 15.89 | 11.06 |
| Grain yield (t ha^-1^) | 5.22 | 5.33 | 5.82 | 2.11 | 11.49 | 9.19 |
| Grain N concentration (%) | 2.31 | 2.33 | 2.51 | 0.86 | 8.65 | 7.72 |
| Straw N concentration (%) | 0.62 | 0.65 | 0.69 | 4.84 | 11.29 | 6.15 |
| Grain N uptake (kg ha^-1^) | 120.35 | 124.30 | 145.84 | 3.28 | 21.18 | 17.33 |
| Straw N uptake (kg ha^-1^) | 41.56 | 45.33 | 53.39 | 9.07 | 28.46 | 17.78 |
| NPFP (kg grain/kg N) | 43.49 | 44.44 | 48.49 | 2.18 | 11.5 | 9.11 |
| NAE (kg grain/kg N) | 17.46 | 18.41 | 22.46 | 5.44 | 28.64 | 21.99 |
| PNB (kg N uptake/kg N) | 1.00 | 1.03 | 1.21 | 3.00 | 21.00 | 17.47 |
| NRE (kg N uptake/kg N) | 0.85 | 0.92 | 1.16 | 8.23 | 36.47 | 26.09 |
| IUE (kg grain/kg N uptake) | 43.36 | 42.90 | 39.90 | -1.06 | -7.98 | -6.99 |
| PE | 27.34 | 27.41 | 26.40 | 0.25 | -3.44 | -3.68 |
| *100 g of urea coated with the extract of 20 g (Vn.Fl-20) fresh leaves of *Vachellia nilotica.* NPFP; Nitrogen partial factor productivity, NAE; Nitrogen agronomic efficiency, PNB; Partial nitrogen balance, NRE; recovery efficiency, IUE; Internal utilization efficiency, PE; Physiological efficiency | | | | | | |
